# Radiation therapy for vaginal cancer in complete uterine prolapse with intrauterine adhesion: a case report

**DOI:** 10.1186/s12905-019-0767-5

**Published:** 2019-05-23

**Authors:** Naoya Ishibashi, Toshiya Maebayashi, Mikiko Asai-Sato, Kei Kawana, Masahiro Okada

**Affiliations:** 1 Department of Radiology, Nihon University School of Medicine, 30-1 Oyaguchi Kami-cho, Itabashi-ku, Tokyo, 173-8610 Japan; 2 Department of Obstetrics and Gynecology, Nihon University School of Medicine, Itabashi-ku, Tokyo, Japan

**Keywords:** Brachytherapy, Vaginal cancer, Uterine prolapse, Intrauterine adhesion

## Abstract

**Background:**

We encountered a woman with vaginal cancer that was associated with complete uterine prolapse and complicated by severe intrauterine adhesions. In this case report, we describe the clinical course and successful treatment of this rare condition.

**Case presentation:**

A 78-year-old woman (gravida 10, para 2, abortion 8) with a 10-year history of uterine prolapse presented for evaluation of bleeding from an ulceration on the surface of the irreducibly prolapsed uterus. Biopsy of a mass on her vaginal wall led to a diagnosis of keratinizing squamous cell carcinoma. Her history of eight abortion procedures had resulted in severe intrauterine adhesions, preventing tandem insertion and intracavitary brachytherapy. She was also ineligible for surgery under general anesthesia + chemotherapy because of her advanced age and presence of arrhythmia. Therefore, we devised an extensive treatment plan involving high-dose-rate interstitial brachytherapy. This treatment successfully eliminated the squamous cell carcinoma as confirmed by biopsy with no recurrence or severe late complications.

**Conclusions:**

We found that high-dose-rate interstitial brachytherapy may be a very effective therapeutic strategy for this condition with few adverse effects.

## Background

Among various types of pelvic organ prolapse, uterine prolapse is occasionally observed in parous women and women of advanced age. When mild cases of uterine prolapse are included, the incidence is reported to range widely from 2.9 to 93.6% among women in the general population [1–3]. The literature contains sporadic reports of uterine cervical cancer in women with uterine prolapse [4–7]. The standard treatment options for medically inoperable uterine cervical cancer are pelvic external beam radiation therapy (EBRT) and brachytherapy (BT). We encountered a woman of advanced age with vaginal cancer associated with complete uterine prolapse, for which BT was planned. The patient had a history of several abortion procedures, and the resultant severe intrauterine adhesions prevented insertion of the tandem. Therefore, we planned high-dose-rate (HDR) interstitial BT (ISBT), in which plastic needles were directly inserted under direct visual observation to surround the margin of the ulcerated lesion of the vaginal cancer associated with complete uterine prolapse. After insertion of the plastic BT needles, dummy sources were inserted and computed tomography (CT) was performed. Using a treatment planning system, dose–volume histogram analysis was performed to develop a plan for covering the lesion, and HDR ISBT was administered. Very few reports have described detailed irradiation procedures for radiation therapy (RT) in the treatment of vaginal or uterine cervical cancer associated with uterine prolapse [8–10]. Furthermore, this is the first reported case of this condition complicated by intrauterine adhesions.

## Case presentation

**Fig. 1:**
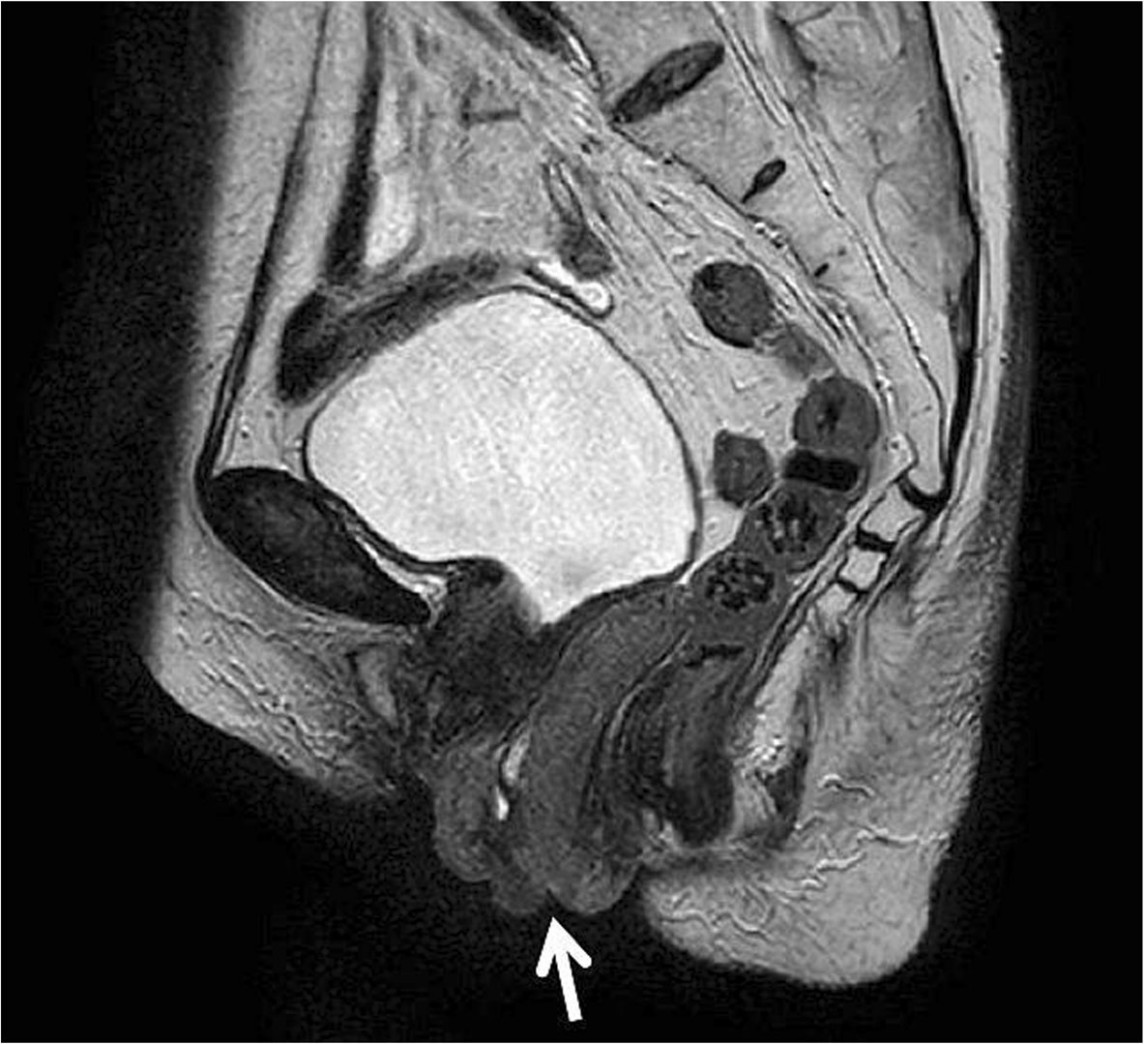
T2-weighted magnetic resonance imaging in the sagittal plane showing a stage IV completely prolapsed uterus defined as eversion of the total length of the lower genital tract. The external cervical os (white arrows) was observed, but no endometrial cavity was detected

**Fig. 2:**
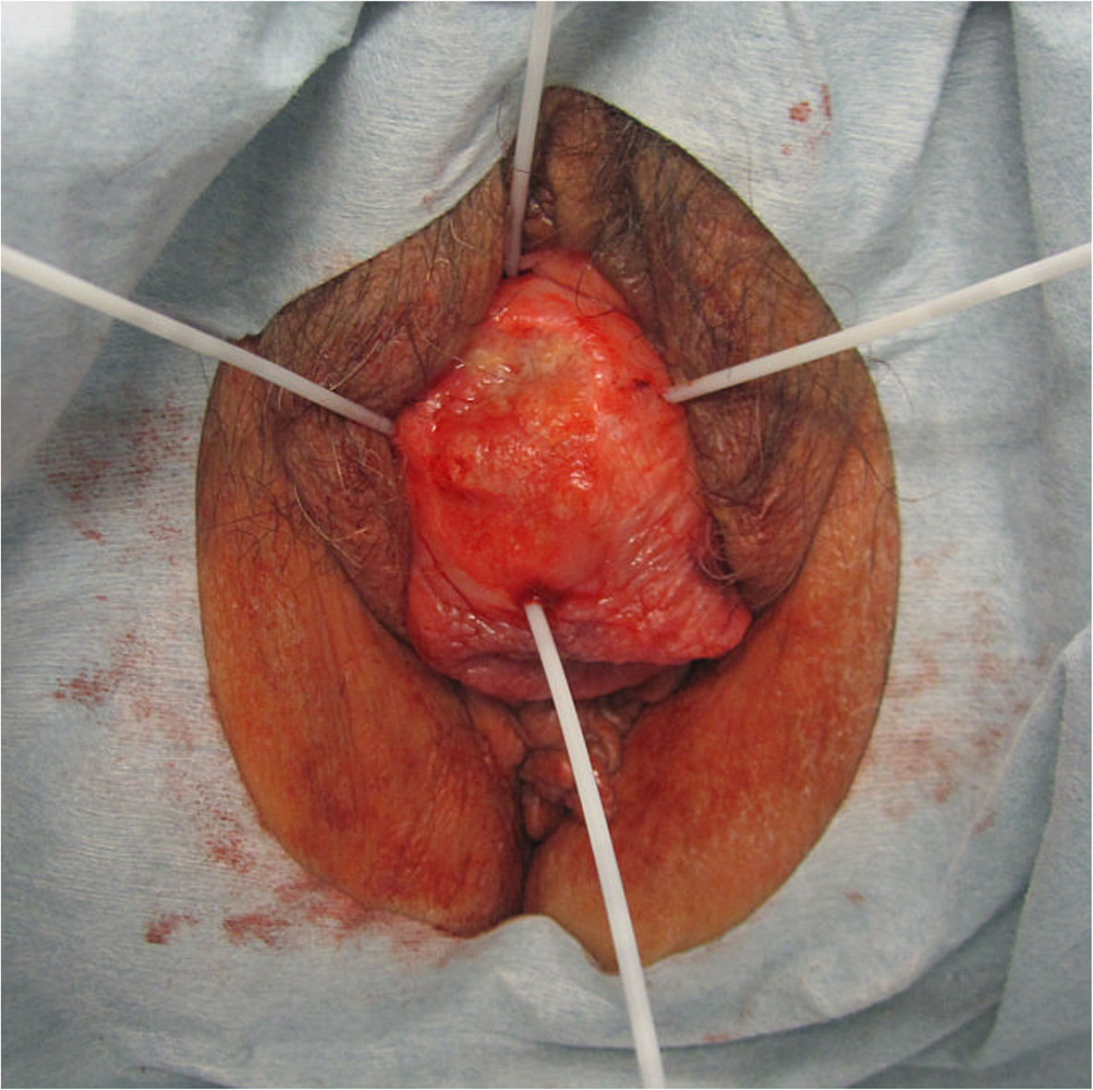
Vaginal cancer with an ulcer in the uterine prolapse (stage IV in the Pelvic Organ Prolapse Quantification System). Four plastic needles were implanted around the ulcer (white arrows)

**Fig. 3:**
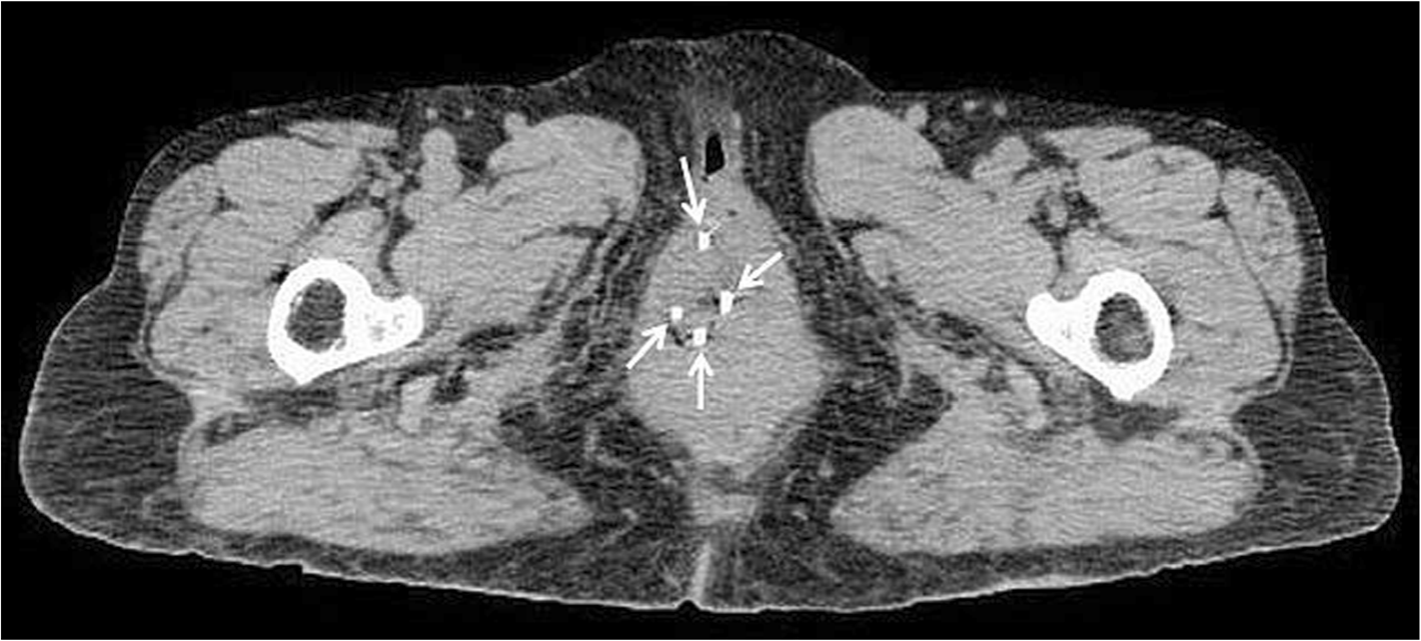
Axial computed tomography scan demonstrating four interstitially implanted dummy sources (white arrows)

**Fig. 4:**
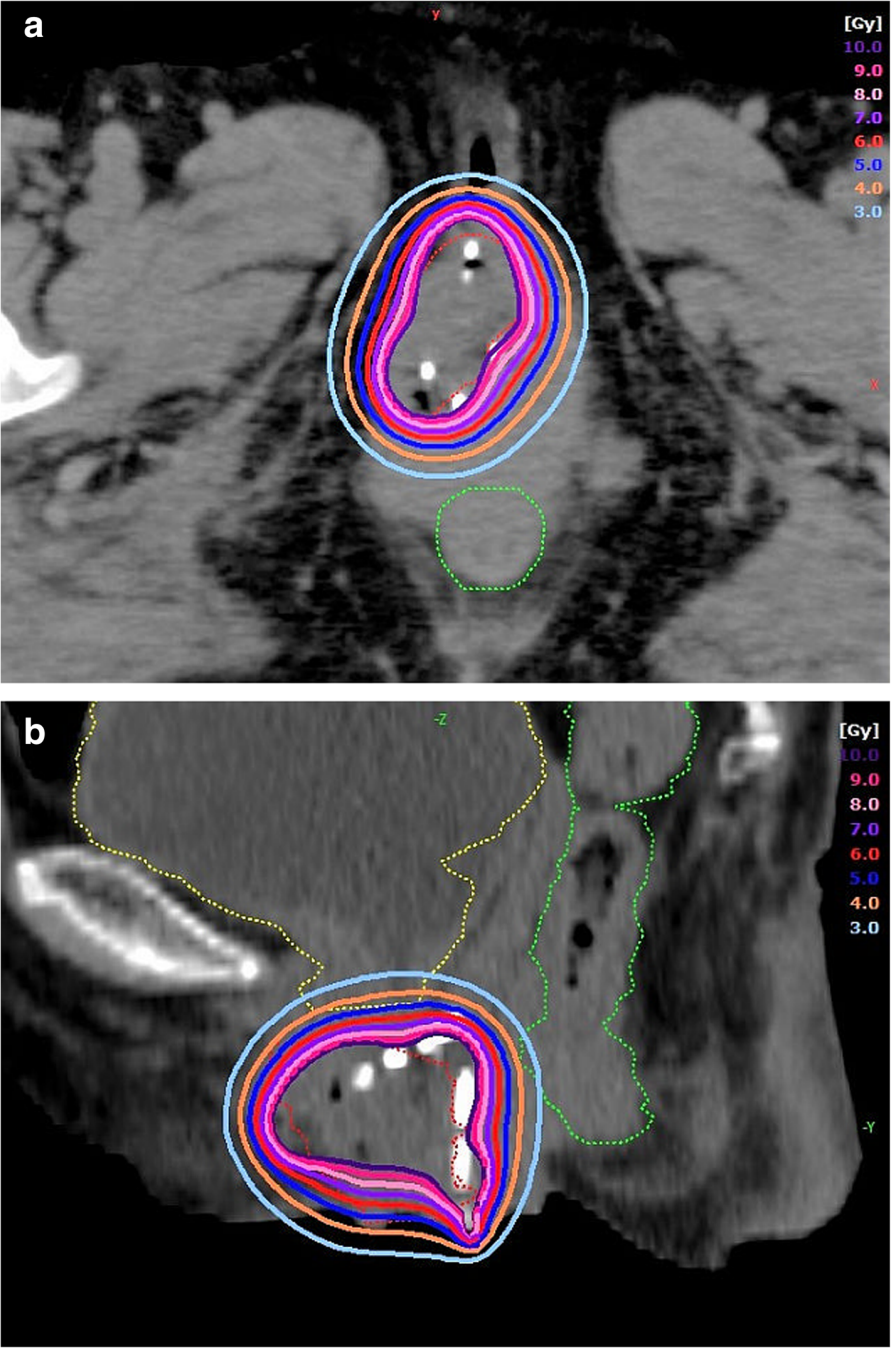
**a** Axial and **b** sagittal computed tomography scans with high-dose-rate interstitial brachytherapy dosimetry from 10- to 3-Gy isodose lines. The the gross tumor volume (red dotted line) was well covered by the prescribed 6-Gy isodose line. The rectum (green dotted line) and bladder (yellow dotted line) are only slightly irradiated

A 78-year-old Japanese woman (gravida 10, para 2, abortion 8) had a 10-year history of uterine prolapse but she had no gynecological examination. She had no smoking history and her body mass index was 19.7. She visited our hospital because of a 1-month history of bleeding from an ulcerated lesion on the surface of the prolapsed uterus. Upon examination, the uterine prolapse appeared as complete eversion of the posterior vaginal fornix and was manually irreducible and classified as stage IV according to the Pelvic Organ Prolapse Quantification System [11]. On the surface of the completely prolapsed uterus, an ulcerated lesion of 3 cm in diameter was observed and a deep-seated induration was felt. A histological biopsy of a mass in the vaginal wall led to a diagnosis of keratinizing squamous cell carcinoma. Magnetic resonance imaging revealed the mass with a depth of 1.6 cm, but the endometrial cavity was not depicted (Fig. 1). The mass was located approximately 3 cm from the cervical os with no sign of invasion to adjacent tissue. According to the International Federation of Gynecology and Obstetrics (FIGO) staging system, the tumor was vaginal cancer staged as I, and a fluorodeoxyglucose positron emission tomography/CT revealed no metastases to the lymph nodes or other organs. Although the diagnosis of this case was vaginal cancer, the main tumor was located in the uterine cervix because of the completely prolapsed uterus. Therefore, we considered that it is practical to develop the treatment plan according to the uterine cervical cancer. Because of the patient’s advanced age and the presence of arrhythmia (paroxysmal supraventricular tachycardia and paroxysmal atrial fibrillation), she was ineligible for a combination of surgery under general anesthesia and chemotherapy; therefore, RT alone was planned. If EBRT had been administered, radiation dermatitis of the genitalia would have been a concern because the prolapsed uterus would be irradiated. Neither a probe nor a tandem could be inserted because of severe intrauterine adhesions due to her history of multiple abortion procedures, also known as Asherman syndrome [12, 13]. The cervical canal was extremely narrowing due to advanced age. Thus, intracavitary BT (ICBT) could not be administered. In addition, the complete uterine prolapse inhibited fixation of an ovoid tandem to the vaginal fornices. Thus, ISBT was planned, in which plastic needles would be directly inserted into the margin of the ulcerated lesion on the surface of the completely prolapsed uterus under direct visual observation. In the BT treatment room, four plastic BT needles (LLA150-K; Eckert & Ziegler BEBIG, Berlin, Germany) were inserted into the margin of the ulcerated lesion to a depth of up to 3 cm under local anesthesia to sufficiently cover the lesion, the depth of which was 1.6 cm as previously measured by magnetic resonance imaging (Fig. 2). Under only local anesthesia, direct insertion of the plastic BT needles caused very little pain. Next, a dummy source for X-ray imaging (LLH02–21 to 24) was inserted into the lumen of each of the four plastic BT needles, and X-ray imaging was performed. The patient was then transferred to the CT room. CT was performed with 2-mm slices, confirming that the BT sources had been inserted around the lesion (Fig. 3). At our facility, HDR ISBT is administered using a ^60^Co remote afterloading system (RALS) (MultiSource; Eckert & Ziegler BEBIG). Thus, these CT scans were uploaded to the RALS treatment planning system (HDR plus; Eckert & Ziegler BEBIG). According to the guidelines established by the Groupe Europeen de Curietherapie and European Society for Radiotherapy and Oncology [14, 15], the gross tumor volume (GTV) and organs at risk (OARs) (i.e., the rectum and bladder) were contoured. The high-risk clinical target volume (HR-CTV) including the whole cervix could not be contoured because the endometrial cavity was not depicted and the whole extent of the cervix was not defined. The GTV was 19.4 cc, and treatment was planned with a dose of ≥6 Gy prescribed as D_90_ of the GTV (minimum dose delivered to 90% of the GTV) (Fig. 4). On another day, the plastic BT needles and dummy sources were reinserted, and X-ray imaging was performed, followed by CT. A similar treatment plan was developed. At our facility, X-ray films alone are used to check the location of radiation sources for BT and develop treatment plans. In this case, X-ray films were obtained after insertion of each plastic BT needle and the dummy sources, and two treatment plans were developed (Table 1). We selected the plan with the dummy sources placed closer to the lesion, according to which HDR ISBT was administered at 6 Gy per fraction twice weekly for a total of eight times. When the location of the dummy sources deviated from the planned sites, the plastic BT needles were reinserted to ensure administration based on one of the treatment plans. The total accumulation dose of HDR ISBT was estimated to be 65.0 Gy as D_90_ of the GTV (equivalent dose in 2-Gy fractions [EQD2]). One month after the completion of RT, the histological biopsy revealed remaining squamous cell carcinoma. Therefore, we performed additional HDR ISBT at 6 Gy per fraction twice weekly for a total of six times until the ulcerated lesion became soft and flat. The final total accumulation dose of HDR ISBT was estimated to be 113.8 Gy as D_90_ of the GTV (EQD2), 20.7 Gy as D_2cm_^3^ of the rectum (EQD2), 36.8 Gy as D_2cm_^3^ of the bladder (EQD2) with α/β = 10 for the GTV and α/β = 3 for the OARs (Table 1). Because of the complete uterine prolapse, the GTV was remote from the rectum and bladder; therefore, the exposure doses to the OARs were extremely low. The only observed acute complication was grade 2 dermatitis in the genitalia (National Cancer Institute Common Terminology Criteria for Adverse Events of 4.03 [16]). One month after the completion of additional HDR ISBT, no residual squamous cell carcinoma was detected in the tissue biopsy. Two months after additional HDR ISBT, as late toxicities, local ulcer lesion with fibrositis was seen in the labium majus and tumor bed in the vaginal wall, and debridement was performed. Three months after additional HDR ISBT, no recurrence or rectal and urinary toxicity had occurred.

**Table 1:** Dose–volume histogram for the gross tumor volume and organs at risk at a prescribed dose of 6 Gy per fraction and the total accumulation dose

Treatment plans	GTV	Organs at risk	
	D_90_	Rectum D_2 cm_^3*^	Bladder D_2 cm_^3*^
1	6.09 Gy	1.81 Gy	2.71 Gy
2	6.03 Gy	1.40 Gy	2.12 Gy
Total accumulation dose	113.8 Gy (EQD2)	20.7 Gy (EQD2)	36.8 Gy (EQD2)

*Abbreviations: D*_*90*_ *of the GTV* minimum dose delivered to 90% of the GTV, *D_*2 cm*_^*3*^ minimum dose delivered to the highest irradiated 2 cm^3^ area, *EQD2* equivalent dose in 2-Gy fractions

## Discussion and conclusions

Although uterine cervical cancer occurring in patients with uterine prolapse has been sporadically reported to date, the incidence rate of this condition is unknown. Based on the information we obtained, the rate is estimated to range from 0.14 to 1.00% [4, 7]. Moreover, no standard treatment for uterine cervical cancer occurring in women with uterine prolapse has been established. Whether this condition should be treated with surgery-based or RT-based therapy remains to be determined [7, 17]. The detailed irradiation procedures used for RT administered for uterine cervical cancer associated with uterine prolapse have been reported in only three cases [8–10] (Table 2), and this is the first reported vaginal cancer of this condition complicated by intrauterine adhesions. Among these previous cases, EBRT alone was administered in one case and a combination of EBRT and ICBT was applied in the other two. Uterine prolapse was spontaneously reduced before EBRT in one case, and reduction was achieved by pessary use and perineoplasty before EBRT in another case [8, 10]. In the third case, the uterine prolapse could not be reduced before EBRT, and radiation cystitis also occurred [9]. When EBRT is administered, reduction of uterine prolapse and hysterectomy are recommended before EBRT to reduce the risks of visceral injury and vesicovaginal or rectovaginal fistulas of the surrounding organs [18, 19]. When BT is administered, however, the exposure doses to the rectum and bladder can be reduced when the uterus remains prolapsed. In fact, the exposure doses delivered to the OARs by HDR ISBT were very low in our case. The standard procedure of RT for uterine cervical cancer is generally a combination of EBRT and ICBT [20]. According to the National Comprehensive Cancer Network Clinical Practice Guidelines in Oncology for Cervical Cancer, BT alone is an option for very early disease, and ISBT is selected in rare cases in which ICBT is not feasible depending on the anatomy or tumor geometry [20]. Furthermore, the American Brachytherapy Society recommends ISBT for cases involving a bulky lesion, a narrow vagina, the inability to enter the cervical os, extension to the lateral parametrium or pelvic side wall, and lower vaginal extension [21]. ISBT was administered in our case because severe intrauterine adhesions inhibited ICBT. HDR ISBT is a therapeutic procedure performed by inserting 5 to 30 needle applicators from the perineum to the cervical cancer under general or spinal anesthesia. This procedure is often combined with ICBT and performed during the later stage or after completion of whole-pelvis EBRT [22–24]. In the present case, we needed to plan RT with the lowest possible toxicity because of her advanced age and arrhythmia; therefore, only HDR ISBT was administered. Thus, neither gastrointestinal nor genitourinary toxicity was observed. Moreover, under local anesthesia only without the need for general or spinal anesthesia, direct insertion of plastic BT needles to the prolapsed uterus caused very little pain. No optimal dose of HDR ISBT for uterine cervical cancer has been determined. However, based on previous reports, the optimal dose per fraction ranges from 4 to 7 Gy for HDR ISBT, and the optimal total D_90_ for HR-CTV (EQD2) ranges from 67.6 to 96.6 Gy when HDR ICBT and EBRT are combined [23–25]. In one study, when stage ≥T3a tumors with a median HR-CTV of 29.8 cc were treated with a total D_90_ for HR-CTV (EQD2) of 80.6 Gy, the local control rate was 83% [23]. In another study, when FIGO stage ≥IIB tumors with a median HR-CTV of 103 cc were treated with a D_90_ for HR-CTV (EQD2) of 67.6 Gy, the local control rate was 80% [24]. Furthermore, when relatively small tumors measuring 2 to 5 cm were treated with a D_90_ for HR-CTV (EQD2) of 89 Gy, the local control rate was 96.9% [25]. Finally, when small tumors measuring ≤4 cm were treated with a total D_90_ for HR-CTV (EQD2) of 69.0 Gy, the local control rate was 96% [26]. In our case, the GTV was 19.4 cc, which is smaller than the HR-CTV described in previous reports. In the first treatment, we treated the total D_90_ for GTV (EQD2) of 65.0 Gy appeared sufficient compared with these previous reports, but biopsy revealed residual squamous cell carcinoma. This first treatment dose may be insufficient if chemotherapy was not performed. We could add HDR ISBT with observation of the treatment effect by biopsy, and the total D_90_ for GTV (EQD2) of 113.8 Gy appeared sufficient. No residual squamous cell carcinoma was detected after completion of the additional HDR ISBT. This additional procedure is advantageous over EBRT. The limitation of our case is the short follow-up period. When vaginal or uterine cervical cancer develops in women with uterine prolapse and intrauterine adhesions, HDR ISBT may be an effective therapeutic strategy with less adverse effects compared with EBRT.

**Table 2:** Cases of cervical/vaginal cancer in patients with uterine prolapse treated by radiation therapy

Patient	Age (years)	Clinical stage (FIGO)	Histology	Prolapse before RT	EBRT field and dose	BT technique and dose	Surgery after RT	RFS (months)	Reference
1	60	IIIB	Large-cell nonkeratinizing squamous cell carcinoma	Reduced under sedation before RT	50 Gy	None	None	2	8
2	73	IIA	W/D keratinizing squamous cell carcinoma	Irreducible	Pelvis 52.2 Gy	Intracavitary HDR 7.5 Gy × 3	Vaginal hysterectomy	60	9
3	72	IIA2	W/D squamous cell carcinoma	Reduced utilizing pessary before RT	Whole pelvis 45 Gy	Intracavitary tandem and ovoids HDR 6 Gy × 5	None	15	10
4	78	I	Keratinizing squamous cell carcinoma	Not reduced	None	interstitial HDR 6 Gy × 14	None	3	our case

*Abbreviations: FIGO* International Federation of Gynecology and Obstetrics, *EBRT* external beam radiation therapy, *BT* brachytherapy, *RT* radiation therapy, *W/D* well differentiated, *RFS* relapse-free survival, *HDR* high-dose-rate

## Abbreviations

BT: Brachytherapy
CT: Computed tomography
EBRT: External beam radiation therapy
EQD2: Equivalent dose in 2-Gy fractions
FIGO: International Federation of Gynecology and Obstetrics
GTV: Gross tumor volume
HDR: High-dose-rate
HR-CTV: High-risk clinical target volume
ICBT: Intracavitary brachytherapy
ISBT: Interstitial brachytherapy
OARs: Organs at risk
RALS: Remote afterloading system
RT: Radiation therapy
